# The Administration of Hypnotics

**Published:** 1907-01-26

**Authors:** 


### SPECIAL ARTICLE.

### The Administration of Hypnotics.

With increasing resources in the drug treatment of sleeplessness comes an ever-increasing responsibility to the physician in the choice of remedies which he shall prescribe. The older notions that pain and sleeplessness were inflictions that should be borne uncomplainingly no longer obtain with any but a very small section of the public, and the ease with which drugs of a harmful or dangerous nature can be procured by the laity is well known and persistently deprecated by the medical profession. At the same time our profession is not wholly absolved from their share of blame in the matter; hypnotics are prescribed in many a case without serious thought of the further effects of the drug or of the temptation to the patient to continue taking it long after the actual necessity has ceased to exist.

To-day, however, with the many additions to the arsenal of therapeutics, and with more exact knowledge of the chemical composition of drugs and of their physiological and pharmacological properties, the evils of self-administration of narcotics may be largely reduced by scientific prescribing on the physician's part. Narcotics are indulged in for two main reasons, their sedative or their stimulating effect. Opium and its derivatives are in the main taken habitually for the sake of their stimulating mental effect, although, amongst Europeans, the habit is usually acquired by the use of the drug as a sedative. Cocaine and Cannabis Indica come into the same category. On the other hand, the new synthetic hypnotics, especially those of the sulphonal or phenacetin group, appear to attract and retain their temptations chiefly for their sedative action; yet in the present state of our knowledge regarding psychological processes it is not justifiable to assume that the habit of taking such drugs depends on a purely negative action, or that the pleasure and gratification consist entirely in the inhibition of conscious mental processes, excepting in such instances as involve the production of insensibility to pain. It is much more probable that the inactivity of certain mental centres permits the increased activity of others, amounting in fact to a stimulus, by diminished resistance or competition on the part of mental processes usually unsuppressed. Some such explanation may very well form the foundation of the habit of taking certain hypnotics whose only action appears to be of a negative character. It is the duty of the medical practitioner to study carefully the effects of the drugs he prescribes in all their bearings and to ascertain the idiosyncrasies, not only physical but mental, of the patients to whom they are administered.

With so large a choice of hypnotics at our disposal it would be unreasonable to adopt the attitude recommended by some, of refusing to prescribe drugs for insomnia in any circumstances, for the physical effects of sleeplessness are so disabling, and the suffering is so real, that to do this merely drives the patient to obtain relief for himself from the chemist's counter, where the want of accurate knowledge on the part of both patient and druggist may lead to the administration of wholly unsuitable and dangerous drugs. Legislation against "counter prescribing" will not prove a remedy against these possibilities. Science must be called in to maintain a successful campaign against ignorance, the physician must acquaint himself with all the latest weapons of pharmacology, and must learn to use them with telling effect against disease, and with safety to the sufferer. The responsibility of choosing a hypnotic to prescribe is no light one and requires an intimate acquaintance not only with the sleep-producing powers of any particular drug, but also with its by- and after-effect, and with its peculiar dangers if any exist. This knowledge of the new drugs which daily present themselves in advertisements from the manufacturing chemists is none too easily acquired.

Disguised under fanciful and proprietary titles, such hypnotics are thrust upon us, armed only too often with false credentials, and eventually prescribed with blind faith by medical men, who have neither the time to question the dogmatic statements of the makers nor the means to identify the composition of the drug. There is at present no handy compendium which can be referred to in order to obtain reliable information on the composition and pharmacology of the thousand and one synthetic hypnotics on the market. Still, in fact, the varieties are not actually so numerous as they seem at first sight. Speaking broadly, there is some active radicle common to each group, whose activity may be assumed to bear some relation to the properties of the particular compound in which it figures. For instance, we are all aware that the opium derivatives may almost without exception be classified according to the morphine each contains. So, too, the phenacetin series depend primarily upon the inclusion of the phenetedin group in their composition. Sulphonal and its allies rest on a particular di-sulphone combination; veronal and hedonal on the fact of being urea compounds of a certain series.

Guided by these considerations, and examining all the proprietary remedies by their aid, it becomes apparent that many a new drug with a cure-compelling title is nothing but an old friend re-christened; that apparently new compounds are nothing but mixtures retaining in their fresh guise all the inertness of their several constituents; or that, though being some other compound than the last advertised, have undergone no substantial variation as regards the radicle upon which their activity depends.

To determine then, judiciously, what hypnotics may advantageously be administered in any given case, a decision may be assisted by a thorough knowledge of the group to which a drug belongs. The fancy title is in itself nothing but a convenience in prescribing. But the chemical formula should be published and accessible. That the medical profession should to a wide extent order and make use of drugs of this character, knowing no more of their composition than the catch-names under which they are introduced to the lay public, goes far to absolve the public when they determine to resort to self-administration. The best method of administration—*e.g.* solution, cachet, etc.—should be stated in each instance; also the time which elapses between the dose and its effects. Every practitioner should be keenly alive to these considerations, and with a view to render some practical help in the course of his practice we give the following particulars of certain new hypnotics. We propose to continue this course as new drugs may call for notice from time to time.

**Neuronal.**

Bromo-di-ethyl-acetamide is one of a large class of soporific drugs whose chief value appears to lie in the treatment of insomnia in the insane; it is, however, of great service in cases of sleeplessness of other origin, particularly in habitual cases where the effect of other hypnotics is beginning to wear off, and where the dose of these is being inordinately increased. As a sedative when pain is present, neuronal has no effect, nor has it any action in preventing the fits of epilepsy. The drug is practically innocuous, it does not depress the patient physically or mentally, and there is said to be no tendency to the formation of a habit. Dosage: 30 to 40 grains. One disadvantage should be pointed out, that many patients either do not react at all or become too easily accustomed to the drug.

**Veronal.**

Of the new hypnotics veronal is perhaps the most reliable in its action and most harmless in its effects. Its chemical composition is di-ethyl-malonyl-urea. In simple insomnia and the sleeplessness of the acute fevers the most satisfactory results have been obtained. Neurasthenia and the milder types of excitement in insanity have yielded readily to its administration. Even after surgical operations, provided there is no great pain, veronal may be used with advantage. The effect of this drug in whooping cough, although so far only limited to comparatively few cases, suggests that it might be more extensively resorted to. Dosage: 5 to 20 grains. Slightly soluble in water, but is best prescribed in powder or tablets. There is practically no tendency to habit-formation.

**Proponal.**

A homologue of veronal with the chemical composition of di-propyl-malonyl-urea, has properties similar to the former but with some noted advantages. The action is more rapid and lasting than veronal, and it has also an anodyne effect. The dose recommended is from 2 to 8 grains, it is freely soluble in dilute alkalies and alcohol, or may be taken in cachets or tablets. In simple insomnia proponal is most efficacious, sleep coming on within half an hour and lasting six to nine hours. In insomnia of the insane proponal may be safely ordered. As an anodyne, provided the pain is not very severe, this drug may procure sound sleep. In hysteria, hypochondriasis, and neurasthenia, it is no less valuable. It is free from unpleasant effects and leaves little or no after depression in moderate doses.

**Hypnal.**

A compound of antipyrine with chloral hydrate, is a powerful hypnotic, combining the properties of its main constituents, but possessing also their disadvantages as a cardiac depressant. But in cases of delirium tremens and acute mania it may prove of great service, and it has been given in some cases of chorea with good results. In simple insomnia the drug should be used cautiously on account of its after-effects and the tendency to habit-formation. It is readily soluble in water. Dosage: 10 to 20 grains.

**Isopral.**

Trichlor-isopropyl alcohol is chiefly of value as an alternative drug in cases of chronic simple insomnia; it rarely fails and only occasionally loses its effect after prolonged administration. Objectionable secondary effects have not been reported, although the physiological action shows isopral to possess to some extent cardio-depressant properties, and also the tendency to cause gastric irritation on an empty stomach. The taste is very unpleasant, but the suggestion has been made of producing hypnosis by inunction of an oily solution of isopral into the skin. Dose: 5 to 30 grains.

**Hedonal.**

Methyl-propyl-carbinol urethane is a reliable hypotic in cases of simple insomnia; it is of no use when pain is present, but will allay a certain degree of excitement in the insane when this does not amount to acute mania. The average dose is 15 to 45 grains, though 60 may be reached. The taste is not pleasant, but it is free from after-effects, except that polyuria has been noticed; in fact, all observers agree on the great safety of the drug. Its power does not become diminished after prolonged use, and there is no cumulative action. Habit-formation does not seem to be recognised.

**Amylene Hydrate.**

Di-methyl-ethyl-carbinol has proved very successful in mania (especially morphinomania), also in alcoholic delirium and even in epilepsy after bromides have failed. The dose is 30 to 60 minims, soluble in water and alcohol, or can be given in capsules; it is suitable for rectal, but not for subcutaneous injection.

**Lactophen.**

One of the phenacetin derivatives (para-phenetedin lactate), is perhaps the most successful of the recent synthetic hypnotics. It is particularly applicable to hypochondriacal and neurasthenic insomnia; moreover, it has an almost specific action upon the headache of influenza or migraine. For delirium tremens or maniacal excitement it has little or no value, nor has it any anodyne properties, save perhaps for the lightning pains of locomotor ataxia, where occasionally it produces considerable alleviation. The dose is from 5 to 15 grains, in powders or tablets. Lactophen possesses the dangers common to the phenetedin group of hypnotics—*i.e.*, palpitation and cardiac depression, with the after-effects of low spirits and lassitude; there is also a great tendency to habit-formation.

